# Concomitant Serological and Molecular Methods for *Strongyloides stercoralis* Screening in an Endemic Area of Spain

**DOI:** 10.3390/tropicalmed9090194

**Published:** 2024-08-25

**Authors:** Ana Lucas Dato, Philp Wikman-Jorgensen, Emilio Borrajo Brunete, María Dolores Hernández Rabadán, Hilarión García-Morante, María Adelino Merino Trigueros, José María Saugar Cruz, Elisa García-Vazquez, Jara Llenas-García

**Affiliations:** 1 Internal Medicine/Infectious Diseases Department, Vega Baja Hospital-Orihuela, 03314 Alicante, Spain; hernandez_mdolores@gva.es (M.D.H.R.); garcia_hilarion@gva.es (H.G.-M.); merino_adelina@gva.es (M.A.M.T.); jllenas@umh.es (J.L.-G.); 2 Foundation for the Promotion of Health and Biomedical Research of the Valencia Region (FISABIO), 46020 Valencia, Spain; 3 Internal Medicine/Infectious Diseases Department, Elda General University Hospital, 03600 Alicante, Spain; 4 Department of Clinical Medicine, Miguel Hernandez University of Elche, 03202 Elche, Spain; 5 Microbiology Department, Vega Baja Hospital-Orihuela, 03314 Alicante, Spain; borrajo_emi@gva.es; 6 Laboratory of Reference and Research in Parasitology, Centro Nacional de Microbiología, Instituto de Salud Carlos III, 28222 Majadahonda, Spain; jmsaugar@isciii.es; 7 Biomedical Research Networking Center of Infectious Diseases (CIBERINFEC), Carlos III Institute, 28029 Madrid, Spain; 8 Infectious Diseases Unit, Hospital Virgen de la Arrixaca, 30120 Murcia, Spain; elisagv@um.es; 9 Medicine Department, University of Murcia, 30003 Murcia, Spain

**Keywords:** *Strongyloides*, screening, migrants, immunosuppressed, serology, stool PCR

## Abstract

Strongyloidiasis is a widespread parasitic disease that can be life-threatening in immunosuppressed people. In the Mediterranean basin, autochthonous cases coexist with imported ones. We aimed to assess the utility of different screening methods, along with the frequency of strongyloidiasis and its associated risk factors in migrants and the native population. This cross-sectional study took place from 2019 to 2022 in the area of the Vega Baja Hospital in Alicante, Spain. Screening was performed in people who were immunosuppressed, at risk of immunosuppression, with blood asymptomatic eosinophilia, and in asymptomatic people from highly endemic countries. Screening methods were serological techniques (ELISA), stool parasitological tests (fecal concentration methods and agar plate culture), and a stool molecular test (PCR). Of the 168 participants (62.5% males, 53.0% migrants, 36.3% immunosuppressed, median age 57 years), 14 (8.3%) had confirmed strongyloidiasis, where 6 were confirmed by serology, 4 by PCR, and 4 by both methods. Overall, 9% of the migrants and 7.6% of the native-born patients were infected. Elevated IgE and hemoglobin and Latin American origin were associated with strongyloidiasis diagnosis. Screening with serology alone would have missed 28.6% of cases. We conclude that strongyloidiasis prevalence is high in our population, both in native and migrant groups, and stool PCR is a useful tool to increase case detection.

## 1. Introduction

Strongyloidiasis is a parasitic intestinal disease in humans that is caused by the soil-transmitted helminth *Strongyloides stercoralis*. Considered to be among the most neglected of all neglected tropical diseases [1], strongyloidiasis showed an estimated global prevalence of 8.1% (95% confidence interval [CI] 4.2–12.4%) according to 2017 data, translating to around 613.9 (95% CI 313.1–910.1) million people infected worldwide [2].

Strongyloidiasis is endemic primarily to tropical and subtropical regions; however, autochthonous transmission has also been reported in temperate climates in Europe and elsewhere [2]. The increase in international travel and especially migratory flows in recent years has led to a rise in imported cases, including in Spain [3], where these are mostly diagnosed in migrants from Latin America and sub-Saharan Africa [4]. These imported cases coexist with autochthonous ones [5], which are concentrated in the Mediterranean basin, especially in the regions of Valencia and Murcia, and have mainly been described in older men involved in agriculture [6,7].

The infection is usually acquired by walking barefoot on infected soil. The larvae penetrate the skin and enter the venous circulation, migrating to the lungs and then to the small intestine, where the larvae develop into adult females, which reproduce asexually and release eggs into the gastrointestinal tract. The eggs then hatch into non-infectious rhabditiform larvae and are excreted in stool [8]. Some rhabditiform larvae become filariform larvae in the large intestine before being excreted. As such, they are capable of reinfecting the host by invading the intestinal wall or the perianal skin. This autoinfection cycle, peculiar to this nematode, can cause persistent (even lifelong) infections if not adequately treated [8].

In most cases, strongyloidiasis remains asymptomatic or produces only blood eosinophilia, while mild symptoms may also present in the skin (rash, pruritus, *larva currens*), digestive tract (intermittent or persistent diarrhea, abdominal pain), or respiratory system (chronic cough, dyspnea). However, in the presence of immunosuppression, for instance, due to hematological malignancy, organ or bone marrow transplantation, immunosuppressive treatments (mainly corticosteroids), or human T-lymphotropic virus type 1 (HTlV-1) infection [9], the cycle of autoinfection can be amplified with larval proliferation, leading to life-threatening complications such as hyperinfection syndrome or disseminated strongyloidiasis [10].

Despite the importance of promptly detecting *S. stercoralis* in immunocompromised patients, very few studies have investigated strongyloidiasis in high-risk groups. The primary aim of this study is to evaluate the implementation of an *S. stercoralis* screening program using serological, traditional, and molecular parasitological methods and to analyze the factors associated with such infestation in both migrants and the native population. We also aim to study the utility of strongyloidiasis screening tools in immunosuppressed and immunocompetent patients.

## 2. Materials and Methods

### 2.1. Study Design

This observational cross-sectional study took place in the catchment area of the Vega Baja Hospital in Alicante, Spain, a health department serving around 166,000 inhabitants [11]. Patients were recruited from April 2019 to September 2022.

The patients were opportunistically recruited from outpatient clinics and the internal medicine ward.

### 2.2. Inclusion Criteria

The inclusion criteria followed evidence-based guidelines [12] and included the following:

1. Patients aged 18 years or older and born in a foreign country endemic for strongyloidiasis (in Africa, Asia, Latin America, or Southeast Europe), whether immunocompetent or immunosuppressed.
2. Autochthonous patients (born in Vega Baja region or residents in this area >30 years) aged 45 years or older, who met the following criteria:
  a Were immunosuppressed or at risk of immunosuppression because of pharmacological treatment (corticosteroids, biologics, or other immunosuppressive drugs) or disease (HIV infection, underlying hematological neoplasm, hematological or solid organ transplant);
  b Had blood eosinophilia of unknown origin.

The age cutoff of 45 years in native patients was decided arbitrarily based on previous studies in our area and in other areas of the Valencian Community [6,7].

Risk of hyperinfection was defined as having any of the following conditions on enrolment: (i) treatment with corticosteroids at a cumulative dose greater than 700 mg in the last three months or the equivalent of 20 mg/day of prednisone for at least 14 days; (ii) treatment in the previous six months with cytotoxic drugs (chemotherapy, alkylating agents [e.g., cyclophosphamide, chlorambucil, melphalan], and antimetabolites [methotrexate > 0.4 mg/kg/day, 6-mercaptopurine > 1.5 mg/kg/day, azathioprine > 3 mg/kg/day, 5-fluoruracil]) or biologics (anti-TNF, rituximab, and other cellular or humoral immunosuppressive drugs); (iii) bone marrow or solid organ transplantation; (iv) active, stage IV solid neoplasia or active hematological malignancy; (v) radiation therapy in the previous six months; (vi) hypogammaglobulinemia; or (vii) HIV infection with a CD4 lymphocyte count below 350 cells/mm^3^.

Patients were considered at risk of immunosuppression if they had a disease that could require immunosuppressant treatment, had HIV with a CD4 lymphocyte count of 350 cells/mm^3^ or more (for potential deterioration of their status in the case of non-retention in care or treatment interruption), were candidates for inclusion on a transplant list, or had a solid neoplasm in low advanced stages without active chemotherapy or radiotherapy treatment.

Eosinophilia was defined as 500 eosinophils/mm^3^ or more.

### 2.3. Study Procedures

All participants were interviewed using an epidemiological and clinical questionnaire that elicited information on sociodemographics (age, gender); involvement in agricultural activities; access to sanitation and hygiene; history of parasitosis; and gastrointestinal, respiratory, or cutaneous symptoms. Migrants were also asked about their country of origin, duration and place of residence in Vega Baja, and visits to their home country. The autochthonous group was asked about their place of birth/residence in Vega Baja, visits to endemic countries, and duration of travel.

### 2.4. Microbiological Studies

After inclusion, each patient provided serum samples for analysis, including complete blood count, IgE determination, and *S. stercoralis* serology (*Strongyloides* IgG ELISA, DRG Instruments Gmbh, Marburg, Germany), along with fecal samples from three separate days in clean plastic containers for coproparasitological study and a fresh stool sample for culture of *Strongyloides* larvae, fresh examination, and DNA detection by real-time polymerase chain reaction (RT-PCR).

The serological study was carried out at the National Center for Microbiology (Health Institute Carlos III, Majadahonda, Madrid, Spain) using a non-automated IVD-ELISA technique that detects IgG antibodies against raw antigen of filariform larvae (DRG Instruments Gmbh, Marburg, Germany). The optical density (OD) index was calculated by dividing the optical density of each sample by the cutoff value. OD values over 1.1 were considered positive; between 1 and 1.1, indeterminate; and less than 1, negative. Microscopic stool examination was performed using the Mini Parasep SF Alcorfix system. The *Strongyloides* culture was seeded in Mueller Hinton agar, incubated at 28 °C, and observed for five days in search of sinuous trajectories. Coproparasitological and fecal cultures were performed at the microbiology laboratory of Vega Baja Hospital. Molecular detection was performed to detect the presence of the parasite in a qualitative real-time PCR (qPCR) assay. Stool samples for RT-PCR were stored at −20 °C until processing. DNA extraction was performed using the QiaAmp DNA stool Mini kit (Qiagen, Hilen, Germany) following the manufacturer’s instructions. For qPCR, *S. stercoralis*-specific primers targeting the 18S ribosomal subunit, as described by Saugar et al. [13], were used. Amplification and detection were carried out using a Corbett Rotor-Gen 6000 RT-PCR cycler (Qiagen Corbett, Hilden, Germany), as described by Saugar et al. [13].

Confirmed strongyloidiasis was defined as positive serology (ELISA) with an OD index of 2.0 or more [14] or a positive result based on parasitological methods (coproparasitological examination, agar plate culture, or PCR stool). Isolated positive serologies with an OD index of 1.1 to 1.9 were considered possible cases.

### 2.5. Patient Treatment

Once microbiological samples were collected, all newly diagnosed patients were treated with one or two doses of ivermectin 200 µg/kg, except in cases of hyperinfection, which were treated with repeated doses according to protocol. In cases where treatment could not be deferred because of immunosuppression, suspected hyperinfection syndrome, or disseminated/complicated strongyloidiasis, treatment was initiated at the screening visit without microbiological confirmation.

### 2.6. Statistical Analysis

For the descriptive analysis, categorical variables were expressed as absolute and relative frequencies. The normality of continuous variables was assessed by the Kolmogorov–Smirnov test and non-parametric variables were described as medians and interquartile ranges (IQRs). In the univariable analysis, categorical variables were compared using the χ^2^ test, and continuous variables by means of the Mann–Whitney U test. In the multivariable analysis, a logistic regression was undertaken using a backwards stepwise selection method among variables yielding *p* < 0.10 in the univariable analysis, plus those considered clinically significant. The quality of the resulting model was assessed using the Hosmer–Lemeshow test and Akaike information criterion, and the discriminatory ability using receiver-operating characteristic (ROC) curves. The significance level was established at *p* < 0.05.

## 3. Results

### 3.1. Cohort Characteristics

**Figure 1:**
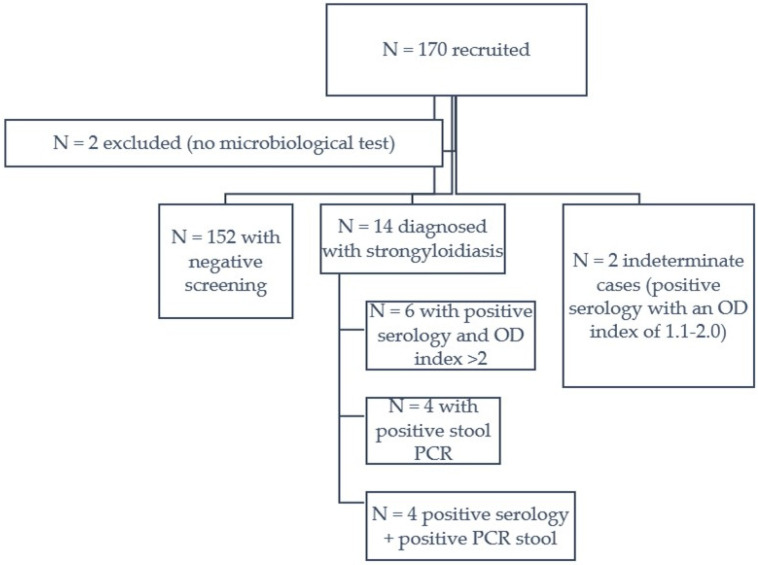
Flow chart for participant recruitment.

In total, 170 patients (n = 80 autochthonous, n = 90 foreign) were recruited from January 2019 to September 2022, and 2 were excluded (n = 1 autochthonous, n = 1 foreign) for not having a Strongyloides serology or a parasitological test performed (Figure 1). The final sample of 168 had a median age of 57 (IQR 45–68) years and was predominantly male (n = 105, 62.5%).

Table 1 summarizes participants’ sociodemographic and clinical characteristics. The foreign participants came mainly from North Africa (n = 41, including 32 from Morocco and 8 from Algeria), South America (n = 31, including 12 from Bolivia and 8 from Ecuador), and Central America (n = 8), and they had resided in Spain for a median of 15.6 years (IQR 5.0–20.6) years. Only eight (10%) autochthonous patients had traveled to endemic countries. A high percentage of patients (n = 123, 73%) had had contact with agriculture.

In total, 14 patients were diagnosed with confirmed strongyloidiasis (six autochthonous and eight migrants). Two additional patients were considered as having possible strongyloidiasis (Figure 1). None of the autochthonous confirmed cases had a relevant travel history.

Gastrointestinal symptoms were reported by 28% of the participants, mainly diarrhea (12.5%), while 23% had respiratory symptoms and 21% had dermatological manifestations. There were no statistically significant differences in the symptoms between those who had confirmed strongyloidiasis and those who did not (Table 2).

There were no cases of hyperinfection syndrome or disseminated/complicated strongyloidiasis. Four patients were treated empirically before having serological and molecular results, mostly for non-delayable immunosuppression; one of them had a positive screening with confirmed strongyloidiasis (case 7 in Table 3).

### 3.2. Factors Associated with Strongyloidiasis Diagnosis

The prevalence of strongyloidiasis varied by place of origin (Table 1); half of the diagnosed patients were from Latin America (odds ratio [OR] 3.77, 95% CI 1.04–13.64; *p* = 0.02), whereas none were from North Africa (0%, OR 0.00, 95% CI 0–0.88, *p* = 0.022). Confirmed strongyloidiasis was also associated with having other parasitosis as well as an elevated eosinophil count, hemoglobin, IgE, and, in people with HIV, higher CD4 cell counts (Table 1).

In the multivariable analysis (n = 168), factors independently associated with confirmed strongyloidiasis were elevated IgE (OR 8.47, 95% CI 1.98–41.64; *p* = 0.004), hemoglobin >13.6 g/dL (OR 2.00, 95% CI 1.31–3.34; *p* = 0.032), and Latin American origin (OR 17.67; 95% CI 2.08–429.01; *p* < 0.001). With these parameters, the area under the ROC curve showed a value of 0.88 (95% CI 0.79–0.97), and the Hosmer–Lemeshow test yielded a goodness-of-fit value of 0.47. Variables eliminated from the model through backwards stepwise selection were sex, African origin, type of footwear, type of WC, and eosinophilia. Table 3 shows the characteristics of patients with confirmed strongyloidiasis.

### 3.3. Microbiological Results and Diagnostic Accuracy

The serology results were positive for strongyloidiasis in 12 (7.1%) cases (Table 4) as follows: 9 (5.4%) with an OD index of 2.0 or more and 3 (1.8%) with an OD index of 1.1 to 1.9. Although 130 patients provided stool samples for fresh examination and culture, none were positive on direct stool microscopy or fecal culture. However, stool PCR was positive in 8/129 patients (6.2%).

Of the 14 patients with confirmed strongyloidiasis, 6 were by positive serology alone with an OD index > 2.0, 4 by a positive PCR, 3 by positive serology with an OD index > 2.0 plus PCR, and 1 by positive PCR plus positive serology with an OD index of 1.1 to 2.0 (Table 4). Two additional patients were considered as having possible strongyloidiasis due to positive serology alone with an OD index of 1.1–2.0.

The frequency of confirmed strongyloidiasis in our sample was 5.4% using serology and 8.3% using serology plus stool PCR. Of the 61 immunosuppressed patients, 4 had confirmed strongyloidiasis, where 2 had a serology with an OD index > 2.0 and a positive PCR and 2 had only a positive stool PCR. Of the 107 immunocompetent patients, 10 had confirmed strongyloidiasis, where 7 had a serology with an OD index > 2.0 (1 also had a positive PCR), 1 had a serology with an OD index 1.1–2.0 and a positive PCR, and 2 had only a positive stool PCR.

On the first visit, only 5/168 patients received ivermectin treatment empirically as follows: 3 for non-delayable immunosuppression, 1 for suspicion of disseminated disease, and 1 based on clinical decision.

## 4. Discussion

Our data showed a high frequency of confirmed strongyloidiasis both in migrants (9.0%) and in the native population (7.6%). Stool PCR improved case detection, as 28.6% of the confirmed cases would have been missed if serology had been the sole screening tool.

Diagnosis of strongyloidiasis is limited by the absence of a gold standard [15]. Stool microscopy is labor-intensive and has low sensitivity for detecting *S. stercoralis* (around 50%) because of intermittent larval excretion and low infectious burden [8]. Sensitivity improves with the use of a *Strongyloides* stool culture and modified agar plate, but it is still under 60% in single samples. Multiple samples have to be examined to achieve adequate sensitivity, but even then, a large proportion of infections will be missed [15]. In our study, traditional parasitological methods surprisingly detected no cases. This may be due to the high workload in the microbiological lab, especially during the coronavirus disease (COVID-19) pandemic, and the lack of a highly experienced parasitologist. Also, asymptomatic cases may have a very low parasite burden, making them especially difficult to diagnose with traditional parasitological methods. While the agar plate culture remains the gold standard for diagnosing strongyloidiasis, combining it with serological and molecular methods can enhance diagnostic sensitivity and specificity [16,17], especially in cases with low parasite burden or in immunocompromised individuals.

Serology might overestimate the burden of disease in endemic countries because of cross-reactivity with other nematode infections [14,15], but this is unlikely in our study as we used an OD cutoff of 2 to improve specificity, and coinfection with other nematodes seems improbable in the autochthonous cases. In a recent study on the diagnostic accuracy of serological tests for *S. stercoralis*, sensitivity and specificity were high, 91.2% and 99.1%, respectively, for IVD-ELISA [14]. Likewise, Schar’s [18] meta-analysis showed that the sensitivity of serology testing ranged from 88% to 98% in community-based studies and 94% to 99% in the hospital setting, justifying its status as the technique of choice for screening asymptomatic migrants [19]. However, in our study, serology as a sole screening tool missed a higher percentage of cases, which may be related to the high percentage of immunosuppressed patients in our sample—a population in whom these methods are less sensitive [20]. In 2001, Schaffel et al. [21] studied the serology of strongyloidiasis in patients with hematological malignancies, reporting a sensitivity of 68%. In their study in 2016, Luvira et al. found even lower sensitivity—42.9%—in immunosuppressed patients, with a specificity of 96.3% [22].

Taken together, these results highlight the need for other screening methods to complement serology in immunocompromised patients, and indeed, the latest guidelines for managing strongyloidiasis in non-endemic countries [12] recommend a combination of serological and parasitological techniques in immunosuppressed patients. Molecular methods have shown modest sensitivity but high specificity [16,17]. In our study, serology plus stool PCR yielded the best results, suggesting that the latter could be an alternative to other fecal-based tests for screening immunosuppressed patients [22], especially in busy but not highly specialized labs, increasing case detection in patients with negative serology.

Our region offers unique opportunities for strongyloidiasis research given the coexistence of autochthonous and imported cases [6]. The overall frequency of strongyloidiasis in our sample was 8.3%, with no statistically significant differences between autochthonous and imported cases. To date, most studies in Spain have been retrospective and focused on imported strongyloidiasis, with prevalence ranging from 0.4% to 46%, according to microbiological techniques, target population, and region [23,24]. A prospective study on Spanish farm workers in the region of Gandia (Valencia) showed a prevalence of 12.4% [25] in high-risk groups compared with 0.3% in the general population. Similar to other studies [26], the median age of confirmed strongyloidiasis cases in our study was 62.5 years, and a high percentage had contact with agriculture. All the autochthonous cases occurred in people over 60 years of age with some contact with agriculture; for that reason and based also on previous studies [6] in our department, we decided to screen only autochthonous patients over 45 years of age. Although there is probably no ongoing transmission in the area, studies should be designed to clarify this aspect.

To our knowledge, only one prospective study on both migrants and at-risk autochthonous people has been conducted to date in Spain [27], specifically in the Canary Islands. Data showed a seroprevalence of 1.1% overall, 2.4% in people awaiting organ transplantation, and 4.8% in those about to start biological agents. However, serology was the only screening method used, so prevalence may be underestimated.

In our sample, positive serology was independently associated with elevated IgE and Latin American origin, which is consistent with previous reports. Elevated serum IgE levels may be a sensitive indicator for strongyloidiasis [28], and a study of imported strongyloidiasis in Spain also reported South America as the most frequent geographical area of origin [4]. In our study, the percentage of migrants from sub-Saharan Africa and Asia was very low, so our results may not be comparable to other areas or countries with different migrant population profiles [18].

Although *Strongyloides* infection is known to produce eosinophilia, we did not observe a statistically significant association between positive serology and eosinophilia, unlike other studies [19,28], although eosinophilia was higher in those screened positive (29% vs. 10%). This was possibly due to the limited sample size and because eosinophilia is intermittent and may fluctuate in chronic infections. On the contrary, we observed higher hemoglobin levels in those who screened positive. We could not find a pathophysiological explanation for this finding; however, anemia is not a frequent manifestation of strongyloidiasis, even in severe cases [29], and higher hemoglobin and red blood cell counts have been reported in at least one previous study [30].

Regarding other parasitosis in patients with positive screens, only Chagas disease coexisted over time with strongyloidiasis in two patients. Its effect on IgE level, eosinophilia, and hemoglobin levels is probably negligible. Eosinophilia has been suggested as a marker of strongyloidiasis coinfection in Chagas disease patients [31].

In patients with HIV, the higher CD4 levels observed in patients diagnosed with *Strongyloides* were unexpected, but this finding is consistent with other studies [32]. Moreover, in the literature, there are published cases of *S. stercoralis* infection as the presenting clinical manifestation of immune restoration syndrome [33,34,35].

Our study has some limitations. First, the sample size was smaller than planned because of difficulties recruiting patients during the COVID-19 pandemic. This, combined with the low frequency of strongyloidiasis, prevents us from drawing definitive conclusions about the laboratory findings and the diagnostic techniques used. Second, the sample was highly heterogeneous, made up of both immunocompetent and immunosuppressed people and autochthonous and migrant populations, making it difficult to generalize the conclusions. Third, there were few included migrants from certain geographical areas (sub-Saharan Africa and Asia), which limits the generalizability of our results to other non-endemic areas with different migration patterns. Fourth, we did not include asymptomatic, low-risk autochthonous patients, so we cannot know the prevalence of strongyloidiasis in that population. Further seroprevalence studies of immunocompetent native populations should be undertaken. Fifth, we did not screen for other helminths that could cause false positives in the serology, such as *Toxocara* spp. However, we used a high OD index cutoff to increase specificity. A positive serology could also be indicative of a past infection, as seroreversion after treatment can take a long period of time; however, this is unlikely in our cohort as none of the patients recalled having received ivermectin before.

The main strength of our study is its prospective nature, with highly accurate data collection and homogeneous diagnostic techniques that were routinely performed for every screened patient.

## 5. Conclusions

Using a combination of serological and molecular techniques for *Strongyloides stercoralis* screening yielded a notable frequency of strongyloidiasis in both high-risk native and migrant populations in the Vega Baja region of Alicante. Elevated IgE and hemoglobin and Latin American origin were associated with strongyloidiasis diagnosis. Screening with serology alone would have missed almost a third of the cases. Stool PCR is useful in increasing case detection, especially in the immunosuppressed population.

## Figures and Tables

**Table 1:** Patient characteristics of the overall cohort and according to confirmed strongyloidiasis.

Characteristics	Total	Strongyloidiasis		OR (95% CI)	*p*
	(n = 168)	Yes (n = 14)	No (n = 154)		
**Demographics**					
Age, years (median, IQR)	57 (45–68)	62.5 (44.75–68.75)	57.0 (45.25–67.75)		0.850
Men (n, %)	105 (62.5%)	8 (57%)	97 (63%)	1.00 (0.00–255.63)	1
**Area of origin (n, %)**					
Vega Baja region (Spain)	79 (47%)	6 (43%)	73 (47%)	0.83 (0.27–2.51)	0.744
North Africa	41 (24%)	0 (0%)	41 (27%)	0.09 (0.01–1.62)	0.103
Sub-Saharan Africa	4 (2%)	1 (7%)	3 (2%)	3.87 (0.38–39.92)	0.255
Europe (other than Spain)	3 (5%)	0 (0%)	3 (5%)	1.49 (0.07–30.34)	0.794
Asia	2 (1%)	0 (0%)	2 (1%)	2.10 (0.09–45.94)	0.636
Latin America	39 (23%)	7 (50%)	32 (21%)	3.81 (1.25–11.66)	0.019
**Occupation (n, %)**					0.608
Farmer	63 (38%)	3 (21%)	60 (38%)		
Rancher	1 (1%)	0 (0%)	1 (0.6%)		
Vegetable packer	9 (5%)	1 (7%)	8 (5%)		
Other	95 (57%)	10 (71%)	85 (55%)		
**Contact with agriculture (n, %)**					1
No	45 (27%)	4 (29%)	41 (27%)		
Main occupation	54 (32%)	3 (21%)	51 (33%)		
Occasional occupation	39 (23%)	6 (43%)	33 (21%)		
Non-occupational contact	30 (18%)	1 (7%)	29 (19%)		
**Swimming in the river (n, %)**	37 (22%)	3 (21%)	34 (22%)	1.06 (0.30–3.73)	1
**Reasons for screening (n, %)**					
Migrants from endemic countries	67 (40.4%)	6 (43%)	61 (40%)	1.16 (0.40–3.39)	1
Immunosuppressed	61 (36.3%)	4 (29%)	57 (37%)	0.73 (0.23–2.30)	0.735
At risk of immunosuppression	33 (19.64%)	2 (14%)	31 (20%)	0.78 (0.19–3.22)	0.861
Blood eosinophilia	20 (11.9%)	4 (29%)	16 (10%)	3.41 (0.70–13.70)	0.067
**Immunosuppressant conditions (n, %)**					
Solid neoplasm/hematological malignancy	41 (17%)	1 (7%)	40 (26%)	0.31 (0.06–1.76)	0.192
Corticosteroid treatment	19 (8%)	1 (7%)	18 (12%)	0.82 (0.14–4.74)	1
Cytotoxic treatment	27 (11%)	1 (7%)	26 (17%)	0.54 (0.09–3.06)	0.473
Biological therapies	21 (9%)	2 (14%)	19 (12%)	1.39 (0.33–5.86)	0.688
Other immunosuppressants	5 (2%)	1 (7%)	4 (3%)	3.72 (0.54–25.60)	0.356
HIV infection	13 (5%)	2 (14%)	11 (7%)	2.50 (0.57–11.02)	0.296
**Clinical symptoms (n, %)**					
Respiratory	39 (23%)	1 (7%)	38 (25%)	0.34 (0.06–1.89)	0.193
Dermatological	35 (21%)	2 (14%)	33 (21%)	0.73 (0.18–2.97)	0.737
Gastrointestinal	48 (28%)	6 (43%)	42 (27%)	2.02 (0.69–5.69)	0.227
**Concomitant diseases (n, %)**					
Cirrhotic chronic liver disease	5 (3%)	1 (7%)	4 (3%)	3.72 (0.54–25.60)	0.356
Chronic kidney disease without dialysis	7 (4%)	1 (7%)	6 (4%)	1.00 (0–255.63)	0.462
Solid neoplasm	26 (15%)	0 (0%)	26 (17%)	0.17 (0.01–2.89)	0.130
Leukemia	2 (1%)	0 (0%)	2 (1%)	1.00 (0.00–255.63)	1
Lymphoma	13 (8%)	1 (7%)	12 (8%)	1.27 (0.21–7.54)	1
Multiple myeloma	5 (3%)	0 (0%)	5 (3%)	0.94 (0.05–17.82)	1
Autoimmune disease	10 (6%)	0 (0%)	10 (6%)	0.47 (0.03–8.52)	1
Inflammatory bowel disease	11 (7%)	2 (14%)	9 (6%)	3.06 (0.68–13.84)	0.230
Other digestive disease	2 (1%)	1 (7%)	1 (1%)	1.00 (0–255.63)	0.160
Skin disease	8 (5%)	0 (0%)	8 (5%)	0.59 (0.03–10.83)	1
Neurological disease	12 (7%)	1 (7%)	11 (7%)	1.00 (0–255.63)	1
Rheumatic disease	9 (5%)	0 (0%)	9 (6%)	0.53 (0.03–9.55)	1
Pneumopathy	19 (11%)	1 (7%)	18 (12%)	0.82 (0.14–4.74)	1
Heart disease	11 (7%)	0 (0%)	11 (7%)	0.43 (0.02–7.68)	0.602
Bone marrow transplant	1 (1%)	0 (0%)	1 (1%)	3.53 (0.14–90.61)	1
Diabetes type 1	2 (1%)	0 (0%)	2 (1%)	2.10 (0.10–45.94)	1
Diabetes type 2	19 (11%)	3 (21%)	16 (10%)	2.55 (0.70–9.38)	0.199
Other	33 (20%)	2 (14%)	31 (20%)	0.78 (0.19–3.22)	1
**Gastroprotective medication (n, %)**	57 (34%)	5 (36%)	52 (34%)	1.13 (0.38–3.40)	0.342
**Immunosuppressant drugs (n, %)**	61 (36%)	4 (29%)	57 (37%)	1.38 (0.44–4.35)	0.539
**Other parasitosis (n, %)**	27 (16%)	6 (42.8%)	21 (13.6%)	4.75 (1.55–14.53)	0.013
**Laboratory tests (first visit), median (IQR)**					
Eosinophils (cells/L)	130 (30–282.5)	230 (115–440)	120 (30–260)		<0.001
Hemoglobin (mg/dL)	12.85 (10.78–14.12)	14.6 (13.2–15.2)	12.17 (10.70–14)		<0.001
IgE (IU/mL)	54.35 (17.3–229.5)	351 (49–1282)	51.5 (15.5–198)		<0.001
CD4 (in HIV+ patients; n = 13)	454 (319–619)	849 (824–874)	428 (195.5–539)		<0.001

OR: odds ratio; CI: confidence interval; HIV: human immunodeficiency virus.

**Table 2:** Clinical symptoms of the global cohort and according to having or not confirmed strongyloidiasis.

	Total	Strongyloidiasis			
Clinical Symptoms	(N = 168) n (%)	Yes (N = 14) n (%)	No (N = 154) n (%)	OR (95% CI)	*p*
**Respiratory**	39 (23%)	1 (7%)	38 (25%)	0.34 (0.06–1.89)	0.192
Cough	35 (21%)	1 (7%)	34 (22%)	0.39 (0.07–2.19)	0.305
Wheezing	18 (11%)	0 (0%)	18 (12%)	0.25 (0.01–4.45)	0.367
Hemoptysis	1 (1%)	0 (0%)	1 (1%)	3.53 (0.14–90.61)	1
**Dermatological**	35 (21%)	2 (14%)	33 (21%)	0.73 (0.18–2.97)	0.737
Urticaria	14 (8%)	0 (0%)	14 (9%)	0.33 (0.02–5.90)	0.609
Pruritus	18 (11%)	1 (7%)	17 (11%)	0.87 (0.15–5.07)	1
**Gastrointestinal**	48 (28%)	6 (43%)	42 (27%)	2.02 (0.69–5.69)	0.227
Chronic diarrhea	21 (13%)	3 (21%)	18 (12%)	2.25 (0.62–8.17)	0.388
Abdominal pain	15 (9%)	3 (21%)	12 (8%)	3.47 (0.92–13.10)	0.115
Dyspepsia	6 (4%)	1 (7%)	5 (3%)	3.02 (0.46–19.98)	0.412
Burning	4 (2%)	0 (0%)	4 (3%)	1.15 (0.06–22.50)	1
Constipation	10 (6%)	1 (7%)	9 (6%)	1.24 (0.15–10.56)	0.844
Nausea	3 (2%)	0 (0%)	3 (2%)	1.49 (0.07–30.34)	1
Vomiting	4 (2%)	0 (0%)	4 (3%)	1.15 (0.06–22.50)	1
Anal itching	1 (1%)	0 (0%)	1 (1%)	3.53 (0.14–90.61)	1

OR: odds ratio; CI: confidence interval.

**Table 3:** Characteristics of patients with confirmed strongyloidiasis.

Case	Sex, Age	Place of Origin	Occupation	Contact with Agriculture	Swimming in the Segura River	Reason for Screening	Immunosuppression	History of Other Parasitosis	Eosinophilia (Cells/µL) IgE level	Diagnostic Method and Results (+/−)	HIV and HTLV-1 Results (+/−)
1	F, 47	Bolivia	Cook	No	No	Migrant from endemic country	None	Chagas	No (440) IgE not requested	Serology + (O.D. 4.4) Stool PCR −	HIV− HTLV-1−
2	M, 44	Panama	Waiter	No	No	Migrant from endemic country	None	No	No (420) IgE 16.20	Serology – Stool PCR +	HIV – HTLV-1 −
3	F, 70	Vega Baja (Spain)	Housewife	No	No	Eosinophilia	None	No	Yes (890) IgE not requested	Serology – Stool PCR +	Not requested
4	M, 62	Bolivia	Furniture upholstery	Occasional occupation	No	Migrant from endemic country	None	Chagas	No (160) IgE 331	Serology + (O.D. 3.31) Stool PCR −	HIV− HTLV-1 −
5	F, 63	Vega Baja (Spain)	Administrative	Non-occupational contact	Yes	Immunosuppressed	Inflammatory bowel disease Biological therapies and corticosteroids	No	No (160) IgE 19.30	Serology – Stool PCR +	HIV− HTLV-1−
6	F, 79	Vega Baja (Spain)	Dressmaker	No	No	Immunosuppressed	Inflammatory bowel disease Biological therapies and corticosteroids	No	No (0) IgE 4.30	Serology – Stool PCR +	Not requested
7	M, 83	Vega Baja (Spain)	Construction worker	Occasional occupation	Yes	Immunosuppressed/ Eosinophilia	Polycythaemia vera, chemotherapy	No	Yes (2490) IgE 2190	Serology + (O.D. 9.68) Stool PCR +	Not requested
8	M, 47	Ecuador	Construction worker	Occasional occupation	No	Migrant from endemic country/ Eosinophilia	None	Amebiasis	Yes (1650) IgE 4770	Serology + (O.D. 15.6) Stool PCR −	HIV− HTLV-1−
9	M, 64	Vega Baja (Spain)	Farmer	Main occupation	No	Eosinophilia	None	No	Yes (1290) IgE 1470	Serology + (O.D. 7.92) Stool PCR −	Not requested
10	M, 43	Ecuador	Farmer	Main occupation	No	Migrant from endemic country	None	Other	No (100) IgE 371	Serology + (O.D. 1.1) PCR +	HIV− HTLV-1 −
11	M, 65	Venezuela	Mechanic	Occasional occupation	No	At risk of immunosuppression	HIV. CD4 799 cells/mm^3^	No	No (100) IgE 1220	Serology + (O.D. 2.48)/ Stool PCR +	HIV + HTLV-1 −
12	F, 35	Honduras	Industrial worker	Occasional occupation	No	At risk of immunosuppression	HIV. CD4 899 cells/mm^3^	Other	No (220) IgE 148	Serology + (O.D. 4.48) Stool PCR −	Not requested
13	F, 78	Vega Baja (Spain)	Vegetable packer	Occasional occupation	Yes	Immunosuppressed	Lymphoma with immunosuppressive treatment	No	No (240) IgE 582	Serology + (O.D. 9.98) Stool PCR +	HIV − HTLV-1 −
14	M, 36	Liberia	Farmer	Main occupation	No	Migrant from endemic country	None	Malaria	No (30) IgE 58.9	Serology + (O.D. 4) Stool PCR −	HIV − HTLV-1 −

Ig: Immunoglobulin. O.D.: optical density index, PCR: real-time PCR. HTLV-1: human T-lymphotropic virus type 1.

**Table 4:** Microbiological data in the overall cohort and in immunosuppressed vs. immunocompetent participants.

Microbiological Techniques for *Strongyloides stercoralis*	Total n/N (%) N = 168	Immunosuppressed n/N (%) N = 61	Immunocompetent n/N (%) N = 107
Serology unequivocal with OD index > 2.0	9/168 (5.4%)	2/61 (3.3%)	7/107 (6.5%)
Serology possible positive with OD index 1.1–2.0	3/168 (1.8%)	1/61 (1.6%)	2/107 (1.9%)
Fresh stool examination positive	0/130 (0%)	0/47 (0%)	0/83 (0%)
Agar plate culture positive	0/130 (0%)	0/47 (0%)	0/83 (0%)
Stool PCR-positive	8/129 (6.2%)	4/42 (9.5%)	4/87 (4.6%)
**Patients with positive microbiological tests**	**N = 14**	**N = 4**	**N = 10**
Patients with only one positive result	10/14 (71.4%)	2/4 (50%)	8/10 (80%)
Serology OD index > 2.0	6/14 (42.9%)	0/4 (0%)	6/10 (60%)
Stool concentration methods	0/14 (0%)	0/4 (0%)	0/10 (0%)
Agar plate culture	0/14 (0%)	0 /4 (0%)	0/10 (0%)
Stool PCR	4/14 (28.6%)	2/4 (50%)	2/10 (20%)
Patients with two positive results	4/14 (28.6%)	2/4 (50%)	2/10 (20%)
Serology OD index > 2.0 + stool PCR	3/14 (21.4%)	2/4 (50%)	1/10 (10%)
Serology OD index 1.1–2 + stool PCR	1/14 (7.1%)	0/4 (0%)	1/10 (10%)

OD: optical density. PCR: real-time polymerase chain reaction.

## Data Availability

All data may be shared and should be requested from the corresponding author.
